# Early auditory processing in musicians and dancers during a contemporary dance piece

**DOI:** 10.1038/srep33056

**Published:** 2016-09-09

**Authors:** Hanna Poikonen, Petri Toiviainen, Mari Tervaniemi

**Affiliations:** 1 Cognitive Brain Research Unit, Institute of Behavioural Sciences, University of Helsinki, P.O. Box 9, FI-00014, Finland; 2 Department of Music, University of Jyväskylä, PL 35(M), FI-40014, Finland; 3 Cicero Learning, University of Helsinki, P.O. Box 9, FI-00014, Finland

## Abstract

The neural responses to simple tones and short sound sequences have been studied extensively. However, in reality the sounds surrounding us are spectrally and temporally complex, dynamic and overlapping. Thus, research using natural sounds is crucial in understanding the operation of the brain in its natural environment. Music is an excellent example of natural stimulation which, in addition to sensory responses, elicits vast cognitive and emotional processes in the brain. Here we show that the preattentive P50 response evoked by rapid increases in timbral brightness during continuous music is enhanced in dancers when compared to musicians and laymen. In dance, fast changes in brightness are often emphasized with a significant change in movement. In addition, the auditory N100 and P200 responses are suppressed and sped up in dancers, musicians and laymen when music is accompanied with a dance choreography. These results were obtained with a novel event-related potential (ERP) method for natural music. They suggest that we can begin studying the brain with long pieces of natural music using the ERP method of electroencephalography (EEG) as has already been done with functional magnetic resonance (fMRI), these two brain imaging methods complementing each other.

In neuroscience, the disclosure of the riddle behind why music has such a strong and unique influence on our mind^1^,^2^ began by studying individual sounds and sound streams^3^. Step by step the musical stimuli and the test settings in the brain laboratories became more complex and involved changing keys, vibrant chords and violated harmonies^4^,^5^,^6^ as well as musical imagination and improvisation^7^,^8^,^9^. More recently, a big leap in the brain research of music was made when Alluri *et al.* studied the cerebral processing of individual musical features extracted from a whole musical piece played in a functional magnetic resonance imaging (fMRI) scanner^10^. Indeed, music as a whole activates the brain widely^11^, but different musical features are processed in different brain regions^10^. Groovy beat travels from the ear into the specific brain structures via different pathways than the sentimental sounds of a violin. While the beat activates movement-related areas, such as the basal ganglia and the supplementary motor area^12^, the calming melodic sound decreases the activation in amygdala thus increasing the activation in other limbic regions^13^,^14^,^15^.

But how are these musical features processed on a shorter time scale, which is out of measurable reach of the temporal resolution of an fMRI? Is there an immediate difference in the processing of musical features between professional musicians and laymen? How is the hearing system tuned to perceive the musical features among professional dancers who also constantly use music in their work and creation? How does a simultaneously presented dance choreography influence to the auditory responses of the musical features? We chose to approach these thrilling questions utilizing the method of event-related potential (ERP) for electroencephalography (EEG). As we have shown before^16^, rapid changes in the musical features of brightness, root mean square (RMS) amplitude, zero-crossing rate and spectral flux during the listening of natural music evokes ERP responses similar to the responses elicited while listening to a series of simple individual sounds.

We chose several long excerpts from the composition Carmen by Bizet-Shchedrin to be presented to professional musicians, professional dancers and a group of participants without any professional background in either music or dance. The musical excerpts were presented as an auditory stimulus, and as an audio-visual entity with a contemporary dance choreography of Carmen. We expected the ERP responses for the musical features to be attenuated and sped up when music was accompanied with concordant dance similar to the results gained by more simple multimodal stimuli^17^,^18^. Since professional background in music has been shown to facilitate the brain processes for individual sounds compared to laymen^19^,^20^, we hypothesized that these kinds of changes would also be detected during continuous music listening. Further, the comparison of dancers and musicians may help in defining whether these changes are influenced by personal history in intense listening of music or in active music-making. Indeed, dancers have a different approach to music than musicians - for dancers the music is a tool for the kinesthetic expression whereas for musicians the music is the essence itself.

## Results

**Figure 1:**
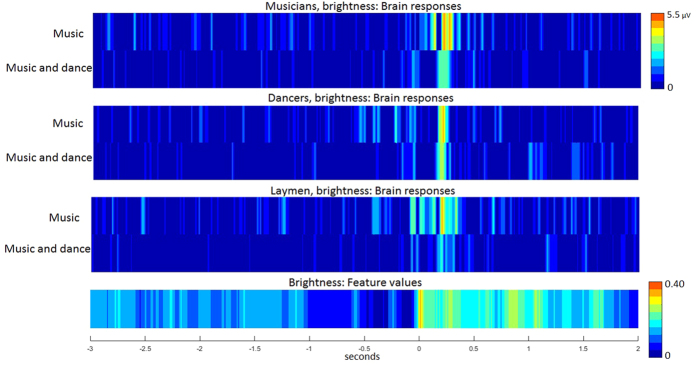
Brain responses of rapid increase in the musical feature brightness in musicians, dancers and laymen during auditory (music) and audio-visual (music and dance) condition. The absolute values of the amplitudes of the EEG epochs are presented over the 16 electrodes in the fronto-central region with the EEG epochs from −3 seconds to +2 seconds from the stimulus onset, and the temporal evolution of the musical feature brightness for the same 5-second time window. The stimulus onset is defined by the end of the Preceding Low-Feature Phase (PLFP) period.

**Figure 2:**
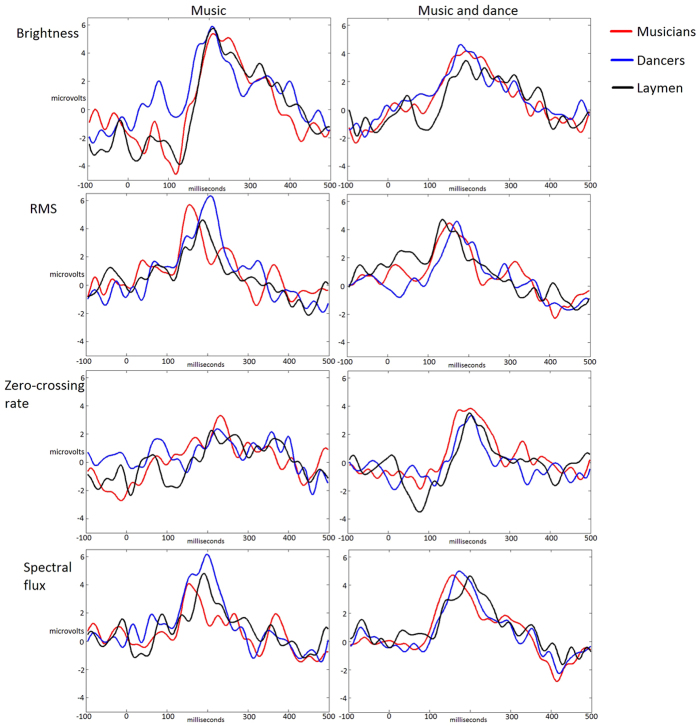
ERPs of the mean value over the averaged signal of 16 electrodes for the rapid changes in the musical features brightness, RMS, zero-crossing rate and spectral flux during the presentation of the auditory stimulus only (music; graphs in the column on the left) and during the stimulus of audiovisual entity (music and dance; graphs in the column on right). In each graph three groups of participants are compared: Musicians, dancers and control group. For brightness, RMS, zero-crossing rate and spectral flux the amount of extracted epochs for each test subject were 9, 8, 8 and 10, respectively, excluding a minimal amount of epochs rejected due to noisy data.

**Figure 3:**
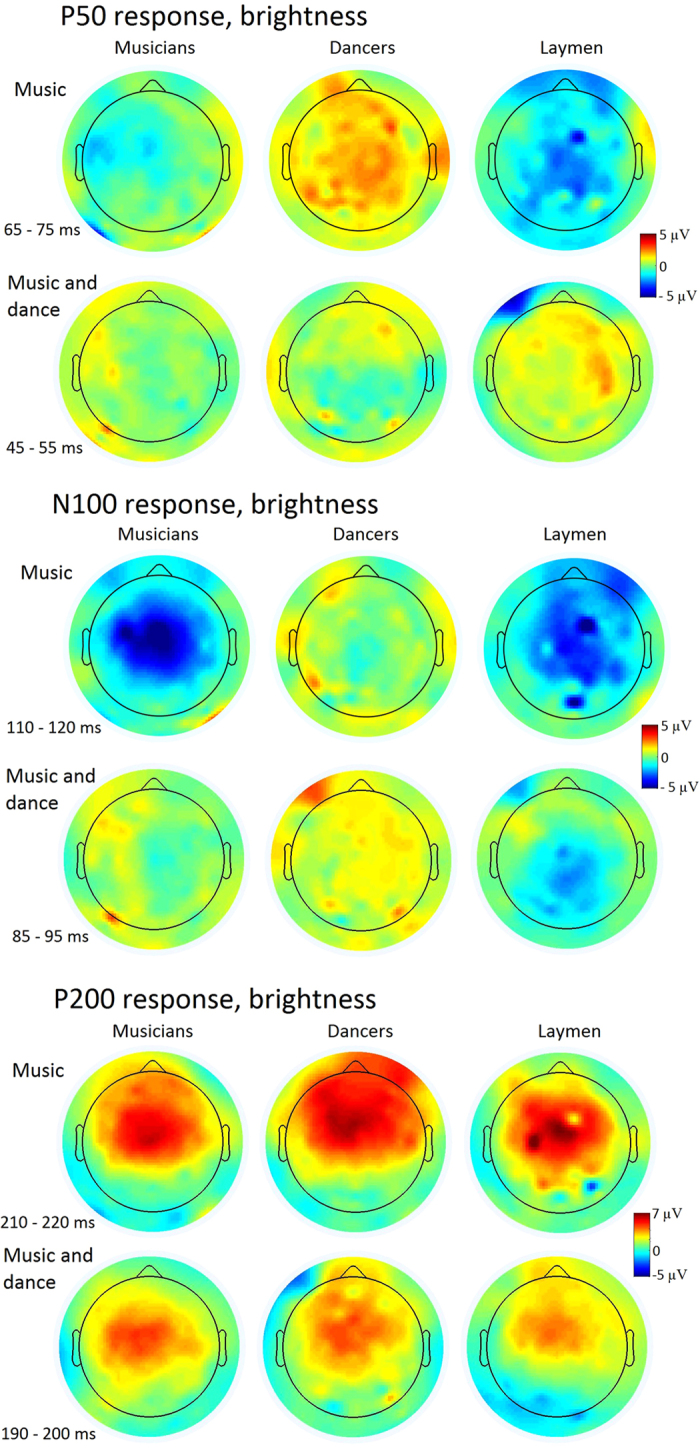
Scalp maps for the P50 (above), N100 (middle) and P200 responses (below) of brightness for musicians, dancers and laymen in auditory (music) and audio-visual (music and dance) condition.

The musical features under interest evoked auditory brain responses resembling those recorded in traditional ERP paradigms. Figure 1 shows the grand-average ERPs in the auditory and audio-visual conditions of the musical feature brightness for musicians, dancers and laymen. Figure 2 shows the recapitulation of the grand-average ERPs in the auditory and audio-visual conditions of brightness, RMS, zero-crossing rate and spectral flux for musicians, dancers and laymen. Scalp maps of the P50, N100 and P200 responses in the auditory and audio-visual condition of brightness for musicians, dancers and laymen are presented in Fig. 3. Statistical evaluation of the data indicated that most but not all of the P50 and N100 responses differed from the zero baseline while all the P200 responses did (see Table 1 for the t-tests of P50 response and Table 2 of N100 response).

In the repeated measures ANOVA, Group (musicians, dancers, control group) was set as the between-subject factor and Modality (auditory, audio-visual stimulus) and Musical feature (brightness, spectral flux, RMS, zero-crossing rate) were set as the within-subject factors.

### P50 response

For the P50 response, neither the amplitude nor the latency showed a significant main effect for the factor Group. For the P50 latency, Modality showed a significant main effect with the Greenhouse-Geisser (GG) adjustment, F(1, 51) = 8.41, pGG = 0.0055 resulting from the latencies of auditory (mean latency 62.5 ms) and audio-visual stimulus (57.1 ms). For P50 amplitude, Musical feature showed a significant main effect, F(3, 153) = 8.11, p = 0.00020 (mean amplitude of brightness 1.79 μV, RMS 3.58 μV, spectral flux 2.04 μV, zero-crossing rate 1.57 μV). For P50 amplitude the Group*Musical feature interaction F(6, 153) = 2.67, pGG = 0.026 was caused by the difference between dancers (2.97 μV) and laymen (1.11 μV), p = 0.014, and between dancers and musicians (1.28 μV), p = 0.030, in the feature brightness revealed by multiple comparison of Group for the musical feature brightness with the critical value of Bonferroni. In addition, P50 amplitude had a significant Musical feature*Modality interaction F(3, 153) = 3.57, pGG = 0.037 rising from the difference of the auditory (1.27 μV) and the audio-visual (2.31 μV) stimulus of brightness, p = 0.047 and of zero-crossing rate, p = 0.0044, with the amplitudes of 2.71 μV and 0.42 μV, respectively, revealed by multiple comparison of Modality with the critical value of Bonferroni. The amplitudes that did not differ significantly, for the auditory stimulus RMS 3.81 μV and spectral flux 2.31 μV, and for the audio-visual stimulus RMS 3.35 μV and spectral flux 1.77 μV.

### N100 response

For the N100 latency the main effects for the factor Modality (F(2, 51) = 11.35, pGG = 0.0014, auditory (98.4 ms) and audio-visual stimulus (86.3 ms)) and for the factor Musical feature (F(3, 153) = 5.69, pGG = 0.0025, the mean latency of brightness 97.5 ms, RMS 85.7 ms, spectral flux 88.1 ms, zero-crossing rate 98.1 ms) were significant. For N100 amplitude, the interaction Group*Musical feature was significant, F(6, 153) = 2.31, pGG = 0.046, rising from the difference between dancers (−2.04 μV) and laymen (−4.69 μV) for the musical feature brightness, p = 0.023, revealed by multiple comparison of Group for the musical feature brightness with the critical value of Bonferroni. With the mean amplitude of −4.43 μV, musicians did not differ significantly from the other groups. Also, for the N100 amplitude, the main effects of Modality (F(1, 51) = 5.85, pGG = 0.019, auditory (−3.17 μV) and audio-visual (−2.41 μV) stimulus) and Musical feature (F(3, 153) = 14.88, pGG = 0.00000014, brightness mean −3.72 μV, RMS −1.60 μV, spectral flux −1.96 μV, zero-crossing rate −3.87 μV) were significant as well as the interaction of Musical feature*Modality, F(3, 153) = 8.44, pGG = 0.00015 caused by the difference of the auditory (−5.15 μV) and the audio-visual (−2.29 μV) stimulus of brightness, p = 0.000036, revealed by multiple comparison of Modality with the critical value of Bonferroni. The amplitudes that did not differ significantly, were for the auditory stimulus RMS −1.90 μV, spectral flux −2.13 μV and zero-crossing rate −3.50 μV, and for the audio-visual stimulus RMS −1.30 μV, spectral flux −1.80 μV and zero-crossing rate −4.25 μV.

### P200 response

For the P200 response, neither the amplitudes nor the latencies differed significantly between the groups. For P200 latency, the main effect of Musical feature (F(3, 153) = 13.80, pGG = 0.0000012, the mean latency of brightness 207.7 ms, RMS 177.5 ms, spectral flux 185.1 ms, zero-crossing rate 206.6 ms) and Modality (F(1, 51) = 6.04, pGG = 0.017, auditory (200.2 ms) and audio-visual stimulus (188.3)) were significant. For P200 amplitude, the main effect of Musical feature (F(3, 153) = 5.65, pGG = 0.0059, the mean latency of brightness 7.33 μV, RMS 7.08 μV, spectral flux 6.80 μV, zero-crossing rate 5.56 μV) and Modality (F(1, 51) = 5.63, pGG = 0.021, auditory (7.08 μV) and audio-visual stimulus (6.30 μV)) were significant as well as the Musical feature*Modality interaction (F(3, 153) = 4.79, pGG = 0.0056 rising from the difference of the auditory (8.16 μV) and the audio-visual (6.51 μV) stimulus of brightness, p = 0.0064 and of RMS, p = 0.0066, with the amplitudes of 7.90 μV and 6.26 μV, respectively, revealed by multiple comparison of Modality with the critical value of Bonferroni. The remaining P200 amplitudes, which did not differ significantly between the modalities, were for the auditory stimulus spectral flux 6.87 μV and zero-crossing rate 5.40 μV, and for the audio-visual stimulus spectral flux 6.72 μV and zero-crossing rate 5.71 μV.

## Discussion

Our results suggest that preattentive processing of changes in timbral brightness of continuous music is improved in dancers compared to musicians and laymen. In addition, brain responses to fast changes in musical features are suppressed and sped up in dancers, musicians and laymen when music is presented with concordant dance. Professional expertise in music can dramatically modulate the auditory processing in the brain^11^,^20^,^21^. Our results gained with continuous polyphonic music extend these earlier results obtained by using simple tones and short sound sequences. Also, our results shed light on how individual characters of a complex sound scene are processed in the brain. Indeed, fast and large changes in particular features of natural music evoke ERP responses corresponding to those evoked by simple sounds. Simultaneous presentation of a dance choreography with music makes our paradigm even more unique in ERP research. In the field of multimodal processing, our paradigm is an upgrade to the earlier studies of ecologically valid audio-visual stimuli^17^,^18^.

Following the interdisciplinary trend of brain imaging using natural stimuli in order to meet the demands of ecological validity^10^,^22^,^23^,^24^,^25^, the music research with ERPs can be upgraded in this respect as well. In addition to the complexity of the physical sound waves, also the human cognition and emotion become much more versatile with the natural musical stimulus. ERP research is necessary to complement the fMRI research because of their fundamental differences in temporal resolution and in the bioelectric origin of the signal.

### ERPs in processing multimodal information

In our study, the auditory N100 and P200 responses were suppressed and sped up in dancers, musicians and laymen during the audio-visual stimulus of a dance choreography compared to the unimodal presentation of the music of the choreography. Previously, Stekelenburg and Vroomen showed how the auditory N100 and P200 responses were suppressed and sped up only if the visual stimulus was synchronized with the auditory event and reliably predicted the sound^17^. As stimuli, they used natural human actions such as the pronunciation of a letter or a hand clap. In their study, N100 amplitude decreased when the visual cue reliably predicted the onset of the sound reducing the temporal uncertainty. In contrast, the P200 amplitude decreased when the content of the visual cue and the sound were coherent, such as the pronunciation of the same letter in voice and in the video. Therefore, N100 likely reflects the multisensory integration related to coherent timing of all the unimodal elements whereas P200 is rather related to the associative and semantic coherence of them^17^. Thus, suggested by the results of the earlier studies^17^,^18^,^26^, dance movement has elements which reliably predict both temporally and associatively fast changes in the musical features reducing the surprise of the sudden change in music. Importantly, neither dancers nor musicians were shown to be more sensitive than laymen to these movement cues suggesting that processes underlying multisensory integration are not modified by the training of music and movement.

In the studies of Stekelenburg and Vroomen^17^,^18^ the audio-visual interaction might have facilitated the auditory processing^27^ by amplifying the signal intensity in the unimodal sensory cortices^28^. Optionally, the visual cue could evoke sensory gating on the auditory cortex^29^ by reducing the novelty and surprise of the sound. The sensory gaiting is shown to suppress P50, N100 and P200 responses in a paired-sound paradigm^30^,^31^. Professional musicians have a reduced paired-sound P50 suppression^32^, yet their N100 is reduced in a manner comparable to that of controls.

### Musical features and group differences

In our study, early cortical processing of music differed in dancers compared to both musicians and laymen. P50 to brightness was larger in dancers than in musicians and laymen. In contrast to the P50, the N100 to brightness in laymen was larger than in dancers, which might be a counter effect of the strong P50 of dancers. In the P200 response the group differences are already diminished.

The processes involved in movement-related imagination could be more active in dancers during their listening to music^33^,^34^, possibly increasing the sensitivity to the fast changes in brightness. Optionally, intense and versatile physical training with music could improve cerebral processes which enhance the early reaction to these changes. Fine temporal changes in music are essential for dancers to create precise rhythmical movement which could, after years of exposure, lead to sensitization in the early auditory processes without concomitant sensitization of the longer-latency responses. Indeed, all large changes of the musical features in the millisecond-scale occur with respect to the temporal structure of music. In addition, pitch, which is an important but not the only factor for brightness, and temporal structure are suggested to be largely integrated in auditory-motor transformations^35^.

Functional integration in the cortico-basal ganglia loops that govern motor control and integration is suggested to be enhanced in dancers compared to laymen^36^. Basal ganglia project not only to the motor cortex but are highly interconnected with widespread areas on the cerebral cortex. Thus, they also play an important role in non-motor cognitive and sensory functions and in a wide range of learning challenges^37^. In vision, cortico-basal ganglia loop participates in action selection in response for a visual stimulus^38^. The auditory cortico-basal ganglia network is less studied but there is evidence for a similar network as in visual domain^39^. Cortico-basal ganglia loop is crucial in the voluntary attentive movements whereas basal ganglia-brainstem loop is involved in the involuntary movements, such as breathing, swallowing and maintaining the body posture. In Parkinson’s and Huntington’s diseases the function of both cortico-basal ganglia loop and basal ganglia-brainstem loop is suggested to be violated^40^. The whole-body movement training of professional dancers seems to modify the cortico-basal ganglia network^36^. When compared to laymen, musicians show modulation on the cortical areas related to sound and movement, especially on the dominant hand of the instrument, and increased connectivity strength in motor-related regions^41^,^42^,^43^. However, it might be the improved cortico-basal ganglia loop of dancers which plays a key role in the enhancement of the preattentive auditory processing of dancers. Similarly to sportsmen, whose motor-related brain areas are sensitized to sports sounds^44^, auditory-motor processes of dancers may be sensitized to musical cues such as rapid changes in brightness. Furthermore, continuous music, which is generally used in dance training, might be a unique stimulus in enhancing top-down controlling of the basal ganglia to the auditory cortex in dancers.

The dance style, in which each dancer was specialized, may have an influence on the early auditory processing of changes in the musical features due to familiarity with the composition or with the musical genre in general^45^. Such specialization of brain functions and structure has previously been shown in musicians^21^,^46^,^47^. Also, a strong background in dance improvisation, and thus possibly enhanced movement imagery during listening to music even without an association to a learned choreography, may have an influence to the preattentive auditory processing by augmenting the sensitivity to the musical cues. The composition used in our study was played with string instruments with occasional percussion. Thus, the musicians specialized in string instruments, might have had enhanced brain responses to the fast changes in the musical features compared to the musicians with biography in non-string instruments^48^.

By means of non-musical stimuli, it could be studied whether this sensitization is related to the musical sounds only or to the auditory information in general. However, it is increasingly common to use non-musical sounds, such as environmental sounds or digital sounds, in the creation of contemporary dance. Familiarity with the composition or with the dance style used in our study could modify the early auditory processing^33^,^49^. Our participants had a versatile background in dance. Thus, a follow-up study in which expertise in specific dance styles are compared, would be important to analyze the effect of familiarity of sound space and of movement language to the early auditory responses.

### Musical features and ERPs evoked by unimodal vs. bimodal stimuli

The musical features were processed differently between the groups of participants as well as between the sensory modalities: During audiovisual presentation of a dance piece, N100 and P200 of brightness and P200 of RMS are attenuated in dancers, musicians and laymen when compared to the auditory presentation. Similarly to our earlier study^16^, the musical feature brightness evoked the strongest ERP responses. Thus, our results suggest that the brain is tuned better to detect the changes in timbral brightness rather than the changes in intensity, harmony or the musical dynamics in general reflected by RMS, zero-crossing rate and spectral flux, respectively. Interestingly, the preattentive P50 response of zero-crossing rate is suppressed but that of brightness enhanced during the audio-visual stimulus when compared to the auditory one. The increased P50 response of brightness is contrary to the results gained with multimodal auditory N100 and P200 responses^17^,^18^. Indeed, the N100 and P200 amplitudes of brightness are suppressed. Possibly, the dance movement anticipates changes in timbral brightness both temporally and associatively. In addition, the intensity-related RMS evokes a suppressed P200 response during the audio-visual stimulus, suggesting that the dance movement predicts associative rather than temporal changes in the intensity of the sound.

Our results propose that long-term activities with music sensitizes the sensory auditory processes despite the music not being produced by oneself. Further research is needed to discover whether this sensitization is due to increased anticipation, attention or some other factors possibly related to the coupling of the auditory and motor systems as discussed above. We did not find differences between the participating groups in the suppression of the ERP responses evoked by a multimodal presentation. In contrast, musical features seem to be processed in the brain along diverging pathways producing variability in the ERP responses of the study groups and of the sensory modalities.

## Conclusions

Our P50, N100 and P200 brain responses suggest that continuous overlapping auditory stimulus such as natural music is processed in the brain at least partly similarly to the simplified sounds traditionally used in ERP research. In contrast, Hasson *et al.* report that, in the visual modality, the brain processes visual stimuli differently in a more ecological setting than in conventional controlled settings^50^. Importantly, the musical features of our study are classified as lower level features evoking bottom-up neural processes. Due to the novelty of the current test paradigm, the musical stimulus could not be optimized beforehand. To evoke clear ERP components in future studies, we recommend to use music which has large changes in the low-level musical features within a short time window. With a replication study of fMRI, Burunat *et al.*^23^ showed constant results in the processing of low-level features whereas the results in the processing of high-level features were not stable. High-level features related to rhythm and melody contour require context-dependent information and evoke top-down processes over a longer time-span^10^,^23^. In addition, the processing of such high-level features may be more sensitive to the state and traits of the listeners, as well as of their background in music^23^. While we analyzed only the post-stimulus cortical processing within a relatively short time window, both further processing of these low-level features as well as the processing of higher level musical features may be different to the conventional simplified sound stimuli. However, our results of cortical sound processing indicate that natural music evokes stronger brain responses than the various traditional simplified stimuli. In fact, with single sounds it has already been shown that with spectrally rich sounds and synthetized sounds mimicking natural instrumental sounds, the brain responses are larger than with pure sinusoidal tones^51^,^52^,^53^. The brain seems to be more sensitive to the stimuli from the real-life environment. Therefore, natural stimuli of continuous music are ideal for applied studies, for example in estimating the depth of coma^54^, the prognosis of vegetative state^55^, comparing the efficiency of medical treatment in psychotic disorders^56^ and estimating the efficiency of expressive therapies such as music and dance/movement therapy.

## Methods

### Participants

20 professional musicians, 20 professional dancers and 20 people without a professional background in either music or dance participated in the experiment. However, two participants from each group were left out from the data analysis since their EEG data lacked several electrodes around the brain area of our interest. Thus, in the groups of musicians and dancers there were 13 female and 5 male participants and in the control group 12 female and 6 male participants. The background of the participants was screened by a questionnaire of music and dance related to both professional and every-day level. Professional background of musicians varied from singing to various instruments, such as piano, violin or saxophone. The professional background of dancers was versatile from ballet and contemporary dance to street dance. Several musicians reported expertise in more than one instrument and several dancers in more than one dance style. The age of the participants ranged from 21 to 31 years (25.4 on average) among musicians, from 23 to 40 years (29.1 on average) among dancers and from 20 to 37 years (25.3 on average) among laymen. Two participants in each of three groups included in the data analysis were left-handed. No participants reported hearing loss or history of neurological illnesses. All subjects gave written informed consent. The experiment protocol was conducted in accordance with the Declaration of Helsinki and approved by the University of Helsinki review board in the humanities and social and behavioural sciences.

### Stimuli

Long excerpts of Carmen composed by Bizet-Shchedrin were used as stimuli. The version of the composition Carmen was performed by Moscow Virtuosi Chamber Orchestra and published by Melodiya, Moscow 1987. Many participants reported being familiar to the composition. The total length of the musical stimulus was approximately 15 minutes, which was cut to 20 trials, the duration of each trial being between 15 and 63 seconds (44.5 seconds on average). Music without visual stimulus, silent dance as well as music and dance as an audiovisual entity were presented to the participants. During the presentation of music only, the participants were advised to listen to the music eyes open although there was no visual stimulus on the screen. The excerpts were chosen from the composition based on their musical and emotional versatility. Some excerpts were musically full and complex whereas the other parts were monotonic and simple. Also, the emotional content varied significantly, some excerpts transmitting a joyful atmosphere, others anger or devastating sadness. The dance choreography presented was based on the contemporary ballet choreographed by Mats Ek. However, the contemporary dancer who performed the dance excerpts for our research purposes, had an artistic freedom to create solo versions to suit her own expression. Thus, the dance choreography was not familiar to any of the participants.

### Equipment and procedure

The stimuli were presented to the participants with the Presentation 14.0 program. Each set of trials contained 20 excerpts of the same sensory modality/modalities and these sets were presented in a random order via a monitor and headphones with the intensity of 50 decibels above the individually determined hearing threshold. Randomization of the presentation order of the stimuli is a standard procedure in experimental psychology which is suggested to reduce the influence of individual differences in other simultaneous cognitive processes. The distance of the monitor from the participant was 110 cm. The participants were advised to listen to the music and watch the dance video as still as possible. The playback of each trial was launched by the researcher. From time to time, between the stimuli, the researcher had a short conversation with the participant via microphone to make sure the participant felt comfortable during the test procedure. The total length of the experiment material was 60 minutes. With pauses and conversations based on the individual needs of each participant, the whole test session lasted about 70–80 minutes.

The data were recorded using BioSemi bioactive electrode caps with 128 EEG channels and 4 external electrodes placed at the tip of the nose, left and right mastoids and under the right eye. The offsets of the active electrodes were kept below 25 millivolts in the beginning of the measurement and the data were collected with a sampling rate of 1024 Hz. The beginning and the end of each musical piece was marked with a trigger into the EEG data.

### Feature extraction with MIRtoolbox

We used MIRtoolbox (version 1.3.1) to computationally extract the musical features. MIRtoolbox is a set of MATLAB functions designed for the processing of audio files^57^ and is used for the extraction of different musical features related to various musical dimensions identified in psychoacoustics and sound engineering as well as traditionally defined in music theory. In addition to the dimensions of dynamics, loudness, rhythm, timbre and pitch also high-level features related to meter and tonality, among others, can be processed. Low-level features are those that are perceived in a bottom-up fashion without a need for domain-specific knowledge. For instance, loudness, pitch and timbre processing automatically recruit sensory mechanisms, and are performed rapidly in very short-time spans. On the other hand, rhythm and melody contour encapsulate context-dependent aspects of music and recruit perceptual processes that are top-down in nature, and require a longer time-span. Since our interest was to study the early auditory processing evoked by fast changes in music, we chose to analyze the following low-level features: Brightness, root mean square (RMS) amplitude, zero-crossing rate and spectral flux. Each one of these features captures a different perceptual element in music.

Brightness was computed as the amount of spectral energy above a threshold value fixed by default in MIRtoolbox at 1500 Hz for each analysis window^57^. Therefore, high values in brightness mean that a high percentage of the spectral energy is concentrated in the higher end of the frequency spectrum. Thus, brightness is influenced by both the pitch of the sound and the characteristic spectrum of the instrument with which the sound is created. Root mean square (RMS) is related to the dynamics of the song and defined as the root average of the square of the amplitude^57^. Louder sounds have high RMS values whereas quieter ones have low RMS values. The zero-crossing rate, known to be an indicator of noisiness, is estimated by counting the number of times the audio waveform crosses the temporal axis^57^. Higher zero-crossing rate indicates that there is more noise in the audio frame under consideration. The noise measured by zero-crossing rate refers to noise as opposed to harmonic sounds rather than to noise as distortion of clean signal. Spectral flux represents the Euclidian distance between the spectral distributions of successive frames^57^. If there is a large amount of variation in spectral distribution between two successive frames, the flux has high values. Spectral flux curves exhibit peaks at transition between successive notes or chords. These musical features were obtained by employing short-time analysis using a 25-millisecond window with a 50% overlap, which is in the order of the commonly used standard window length in the field of Music Information Retrieval (MIR)^58^. Overlapping of windows is recommended in the analysis of musical features to detect fast changes in the features and their possible inactive periods with a precise time resolution.

### Preprocessing

The EEG data of all the participants were first preprocessed with EEGLAB^59^ (version 9.0.2.2b). The external electrodes of the left and the right mastoid were set as a reference. The data were high-pass filtered at 1 Hz and low-pass filtered at 30 Hz.

### Setting the Triggers

The triggers related to the musical features extracted with MIRtoolbox were added to the preprocessed EEG data. In continuous speech, the best ERP-related results are gained when the triggers are set into the beginning of the word^60^,^61^. Long inter-stimulus interval is shown to increase the amplitude of the N100 response^62^. Additionally, strong stimulus intensity has been shown to enhance ERP responses^63^,^64^. Previous knowledge from the individual sound processing was utilized in our study of continuous music, in which the individual sounds are connected to each other in an overlapping and dynamic manner.

Approximately 10 triggers per each feature were set. We used the same MATLAB algorithm for the search of time points with rapid increase of a musical feature as was used in the study of Poikonen *et al.* for defining the time points of the trigger^16^. The algorithm was tuned using specific parameter values adapted to each musical feature. In our study, the time period with low feature values preceding the rapid increase in the value of the musical feature corresponds to the inter-stimulus interval (ISI) of the previous literature. However, in our study, the intervals are not between individual stimuli anymore nor are the intervals completely silent, and thus this ISI-type of period is called the Preceding Low-Feature Phase (PLFP) in this paper.

The length of the PLFP was modified and the rapid increase was required to exceed a value called magnitude of the rapid increase (MoRI). The mean values of all the segments of each one of the 20 sound excerpts and each musical feature were calculated and the magnitude of the change from the lower threshold value V_n−_ to the higher threshold value V_n+_ was defined based on the mean value (MV_n_) in each particular sound excerpt for each musical feature. The largest changes in the musical features were when the V_n−_ remained under −20% of MV_n_ and V_n+_ increased above +20% of MV_n_. The smallest changes in the musical features were when the V_n−_ remained under −15% of MV_n_ and V_n+_ increased above +15% of MV_n_. Valid triggers were preceded by a PLFP whose magnitude did not exceed the lower threshold V_n−_. The length of PLFP with values below V_n−_ was 625 milliseconds minimum and 1 second maximum. In all cases, valid triggers had an increase phase that lasted less than 75 milliseconds during which the feature value increased from V_n−_ to V_n+_.

### Procedure of the ERP analyses

After adding the triggers into the preprocessed data, the data were treated with Independent Component Analysis (ICA) decomposition with the runica algorithm of EEGLAB^59^ to detect and remove artifacts related to eye movements and blinks. ICA decomposition gives as many spatial signal source components as there are channels in the EEG data. Thus, the amount of components was 128 in 22 participants. In the remaining 32 participants, several noisy channels each were removed in preprocessing and therefore less than 128 ICA components were decomposed in them. Typically, 1 to 5 ICA components related to the eye artifacts were removed. Noisy EEG data channels of the abovementioned 32 participants were interpolated. The average number of interpolated channels among these 32 participants was 3.1 channels, the actual number of interpolated channels varying from one per person up to 8 per person. The continuous EEG data were separated into epochs according to the triggers. The epochs started 500 milliseconds before the trigger and ended 1000 milliseconds after the trigger. The baseline was defined according to the 500-millisecond time period before the trigger. To double check the removal of the eye artifacts, the epochs with amplitudes above ± 100 microvolts were rejected.

The statistical analyses were conducted with MATLAB version R2015b. In the statistical analysis, 16 electrodes (B1, B21, B22, B32, C1, C2, C11, C22, C23, C24, D1, D2, D13, D14, D15 and D18 of the 128-channel BioSemi EEG gap) were averaged as one signal. Cz was not included among the averaged channels because it was not recorded from five participants due to a broken electrode. Each participant had only 8–10 trials for each musical feature in each sensory modality due to the need to minimize the duration of an experimental session, which was already 60 minutes long. To improve the S/N ratio of the signal, we averaged the signal over several electrodes.

According to the Shapiro-Wilk test, 75.0% of the P50 responses, 87.5% of the N100 responses and 75.0% of the P200 responses were normally distributed. Thus, the repeated measures ANOVA was used in the statistical analysis.

The repeated measures ANOVA was calculated for both amplitude and latency of the P50, N100 and P200 responses. A time window from 30 ms to 90 ms was chosen for the statistical analyses of the P50 response, a time window from 50 ms to 150 ms for the N100 response and a time window from 100 ms to 280 ms for the P200 response.

## Additional Information

**How to cite this article**: Poikonen, H. *et al.* Early auditory processing in musicians and dancers during a contemporary dance piece. *Sci. Rep.* **6**, 33056; doi: 10.1038/srep33056 (2016).

## Figures and Tables

**Table 1:** P50 response (time window from 30 milliseconds to 90 milliseconds of the stimulus onset).

t-test	t_17_	p
Musicians		
Brightness		
Auditory stimulus	0.63	0.54
Auditory-visual stimulus	3.97	0.0010
RMS		
Auditory stimulus	5.71	0.000025
Auditory-visual stimulus	3.43	0.0032
Zero-crossing rate		
Auditory stimulus	2.75	0.014
Auditory-visual stimulus	1.67	0.11
Spectral flux		
Auditory stimulus	3.40	0.0034
Auditory-visual stimulus	1.74	0.10
Dancers		
Brightness		
Auditory stimulus	4.82	0.00016
Auditory-visual stimulus	3.49	0.0028
RMS		
Auditory stimulus	4.38	0.00041
Auditory-visual stimulus	3.45	0.0031
Zero-crossing rate		
Auditory stimulus	6.12	0.000011
Auditory-visual stimulus	0.28	0.79
Spectral flux		
Auditory stimulus	0.77	0.45
Auditory-visual stimulus	2.06	0.055
Laymen		
Brightness		
Auditory stimulus	0.44	0.66
Auditory-visual stimulus	3.91	0.0011
RMS		
Auditory stimulus	4.28	0.00050
Auditory-visual stimulus	3.91	0.0011
Zero-crossing rate		
Auditory stimulus	3.30	0.0043
Auditory-visual stimulus	−0.13	0.90
Spectral flux		
Auditory stimulus	3.09	0.0067
Auditory-visual stimulus	3.61	0.0021

T-tests over the averaged signal of the 16 electrodes in the fronto-central region for musicians, dancers and laymen in the auditory and audio-visual condition of the musical features brightness, RMS, zero-crossing rate and spectral flux.

**Table 2:** N100 response (time window from 50 milliseconds to 150 milliseconds of the stimulus onset).

t-test	t_17_	p
Musicians		
Brightness		
Auditory stimulus	−5.82	0.000021
Auditory-visual stimulus	−3.00	0.0089
RMS		
Auditory stimulus	−2.32	0.033
Auditory-visual stimulus	−1.43	0.17
Zero-crossing rate		
Auditory stimulus	−4.30	0.00048
Auditory-visual stimulus	−5.90	0.000017
Spectral flux		
Auditory stimulus	−5.78	0.000022
Auditory-visual stimulus	−1.76	0.096
Dancers		
Brightness		
Auditory stimulus	−2.72	0.015
Auditory-visual stimulus	−1.93	0.070
RMS		
Auditory stimulus	−2.34	0.032
Auditory-visual stimulus	−3.30	0.0042
Zero-crossing rate		
Auditory stimulus	−5.00	0.00011
Auditory-visual stimulus	−4.34	0.00044
Spectral flux		
Auditory stimulus	−1.80	0.089
Auditory-visual stimulus	−3.34	0.0038
Laymen		
Brightness		
Auditory stimulus	−7.12	0.0000017
Auditory-visual stimulus	−4.61	0.00025
RMS		
Auditory stimulus	−3.34	0.0039
Auditory-visual stimulus	−1.05	0.31
Zero-crossing rate		
Auditory stimulus	−5.45	0.000043
Auditory-visual stimulus	−6.13	0.000011
Spectral flux		
Auditory stimulus	−3.52	0.0026
Auditory-visual stimulus	−2.36	0.031

T-tests over the averaged signal of the 16 electrodes in the fronto-central region for musicians, dancers and laymen in the auditory and audio-visual condition of the musical features brightness, RMS, zero-crossing rate and spectral flux.
